# Supplementary material for: A light-off response characterised by body contraction and ciliary arrest in Acropora coral larvae
Source: J Exp Biol. 2026 May 15;229(10):jeb252360. doi: 10.1242/jeb.252360 (PMC13245902; doi:10.1242/jeb.252360)
Supplement: Supplementary information [file jexbio-229-252360-s1.pdf]

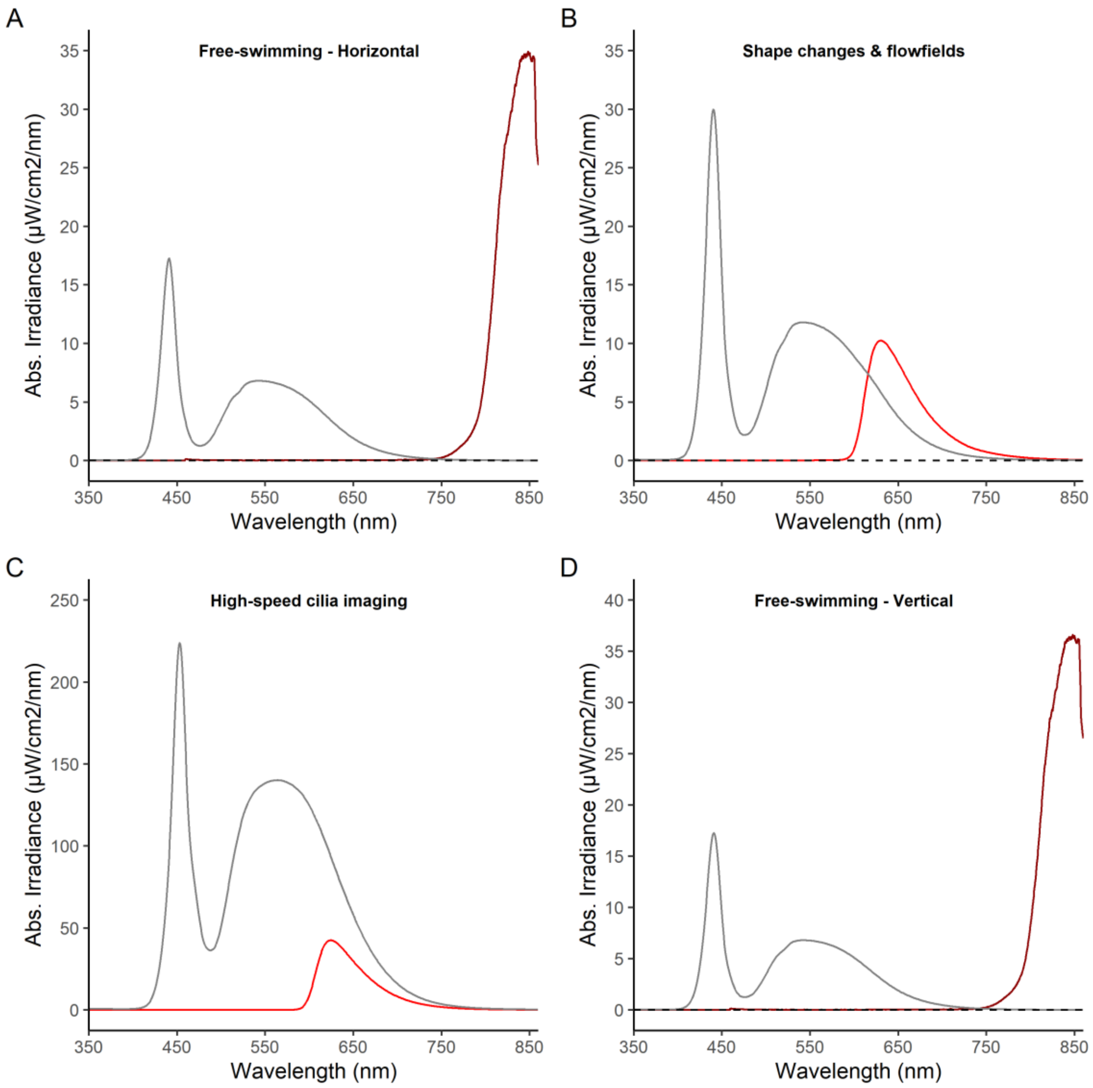

Fig. S1. Absolute irradiance (μW cm^{-2} nm^{-1}) light spectra measured from experimental apparatus. A) Free-swimming experiments using the shallow cuvette to assess horizontal movement. Bright white LED stimulus when on (grey solid line) and off (black dashed line). Solid dark-red line shows the near-infrared ring light, used constantly for camera illumination. **B)** Tethered larvae experiments to assess larval shape changes and flow fields. Bright white LED stimulus when on (grey solid line) and off (black dashed line). Solid red line shows the dim red-filtered illumination used for imaging. **C)** High-speed imaging of tethered larvae to assess ciliary beating. Bright white light stimulus when on (grey line) and when passed through a red filter (red line). **D)** Free-swimming experiments using the tall cuvette to assess vertical movement. Bright white LED stimulus when on (grey solid line) and off (black dashed line). Solid dark-red line shows the near-infrared ring light, used constantly for camera illumination. All spectra measurements were made from the position of the experimental cuvette where the larvae were held.

Journal of Experimental Biology • Supplementary information

Table S1. Wilcoxon rank sum statistical tests (W) comparing swimming speed (mm s^{-1}) in the second half of dark and light time periods for the horizontal free-swimming experiments. Columns 2 and 3 are median swimming speed values for each light condition. The bottom row shows the test result when data from all batches were pooled together.

batch	dark_med	Light-on_med	W-value	p-value	signif.
1	0.050	0.340	3062	6.80 e-15< s^{-1}up>	***
2	0.039	0.241	4141	8.75 e-27	***
3	0.039	0.372	2916	1.55 e-18	***
4	0.044	0.151	4618	1.02 e-11	***
5	0.083	0.212	6730	8.89 e-08	***
6	0.123	0.241	8905	0.0218	*
**pooled**	**0.058**	**0.235**	**183558**	**2.91 e**-63	***

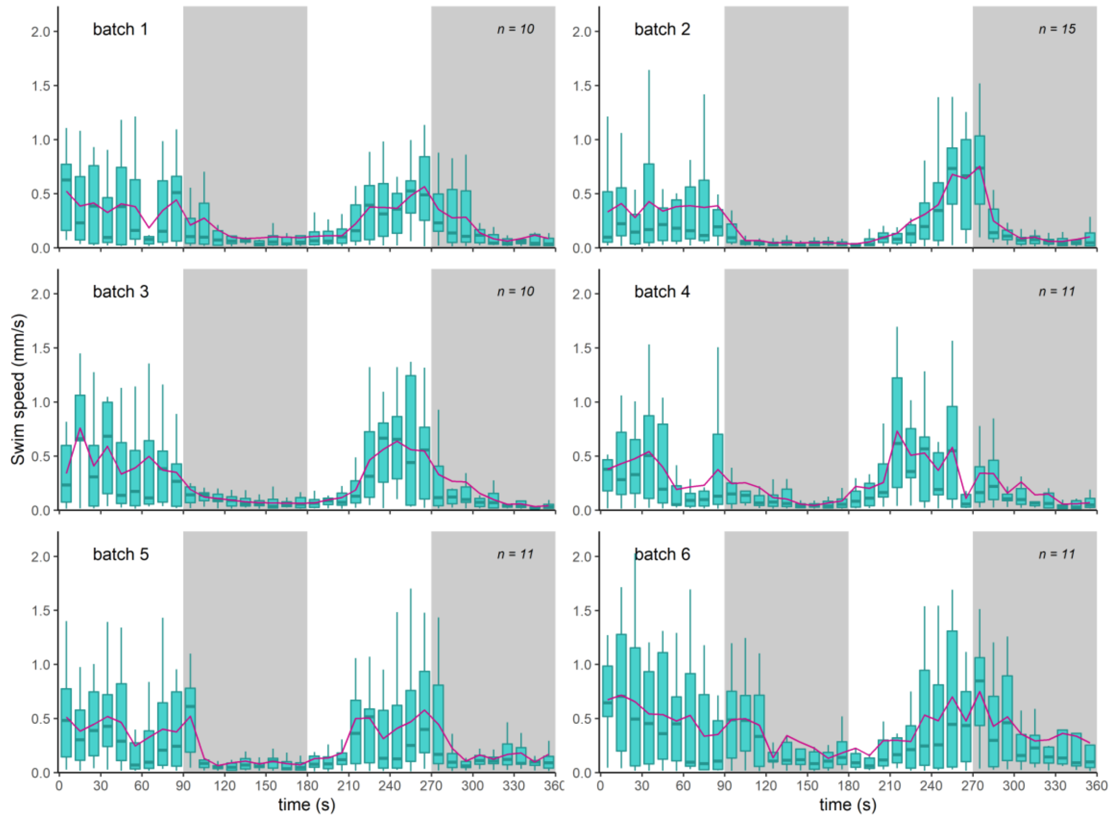

Fig. S2. Swimming speed data (mm s^{-1}) for six batches of larvae in the horizontal free-swimming cuvette experiment. Data are represented as boxplots of the interquartile range with median line and whiskers extending to the most extreme values within 1.5× the interquartile range. Purple line represents mean swimming speed. White areas represent time periods with the light stimulus being on, whereas shaded boxes show the dark time periods.

Table S2. Wilcoxon rank sum statistical tests (W) comparing horizontal displacement (mm s^{-1}) in the second half of dark and light time periods for the horizontal free-swimming experiments. Positive median values in columns 2 and 3 represent movement toward the light source on one side and negative values represent movement away from it. The bottom row shows the test result when data from all batches were pooled together.

batch	dark_med	light-on_med	W-value	p-value	signif.
1	0.019	-0.004	6878	0.4424	-
2	-0.064	0.140	10995	0.0081	**
3	-0.016	0.008	8234	0.7845	-
4	-0.004	-0.005	8397	0.4157	-
5	0.015	-0.038	11484	0.2054	-
6	-0.044	-0.066	10555	0.9939	-
**pooled**	**-0.023**	**-0.004**	**338476**	**0.2727**	**-**

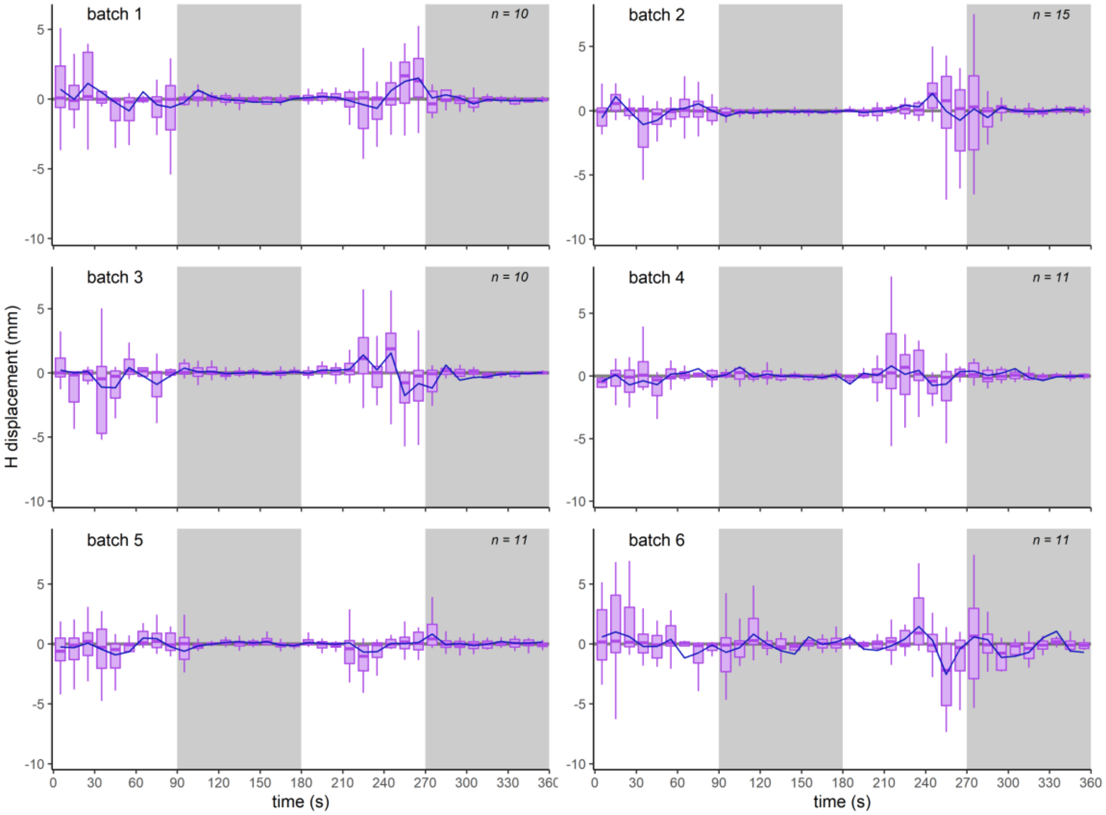

Fig. S3. Horizontal displacement data (mm s^{-1}) for six batches of larvae in the horizontal free-swimming cuvette experiment. Data are represented as boxplots of the interquartile range with median line and whiskers extending to the most extreme values within 1.5× the interquartile range. Dark blue line represents mean swimming speed. White areas represent time periods with the light stimulus being on, whereas shaded boxes show the dark time periods.

Journal of Experimental Biology • Supplementary information

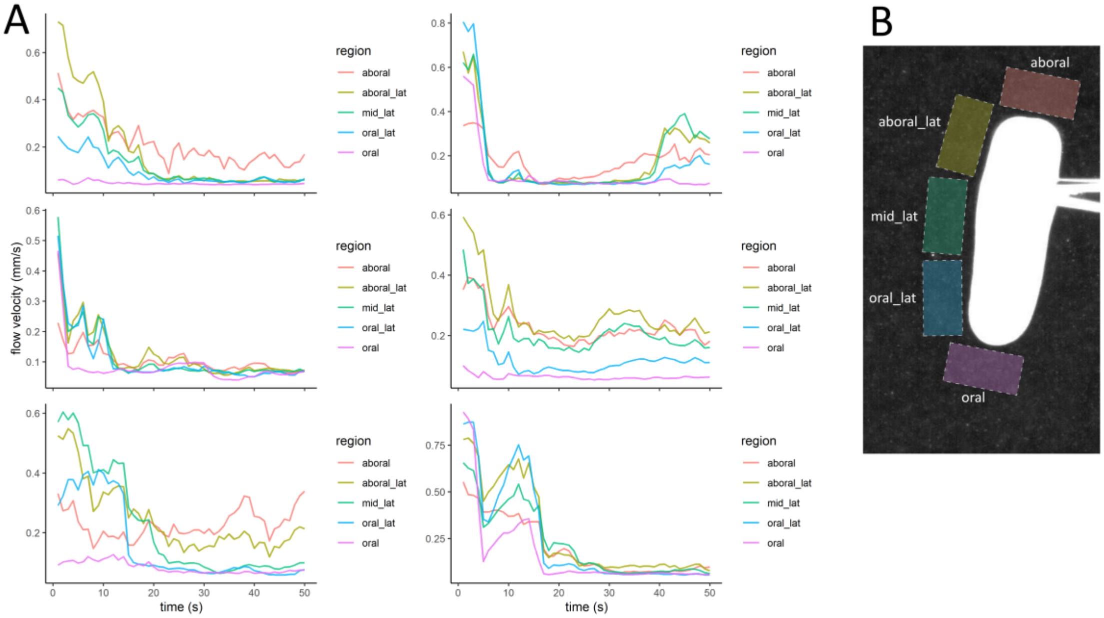

Fig. S4. Spatially resolved flow velocities measured by particle image velocimetry (PIV) around tethered larvae. (A) Flow velocity (mm s^{-1}) within five regions spanning the aboral to oral pole is shown for each larva (one plot per individual). Colours indicate the sampled regions. Time 0 corresponds to the moment when LED illumination was switched off, marking the start of the "dark" period. The first 50 s of this 90 s dark interval are shown to illustrate the light-off response. **(B)** Representative example showing the five spatial regions from which flow velocity was quantified using PIV.

Table S3. Wilcoxon rank sum statistical tests (W) comparing the aspect ratio (length/width) and 2D areas of larval profiles in the second (45 s) half of dark and light time periods. Median values are shown for the light periods for both 2021 and 2022 batches of larvae.

year	dark_med	light_on_med	W-value	p-value	signif.
**Aspect ratio**					
2021	1.35	1.51	1058.5	0.0014	**
2022	1.22	2.15	551	8.50 e-11	***
**2D area**					
2021	234	256	1262	0.0425	*
2022	215	243	716	1.89 e-08	***

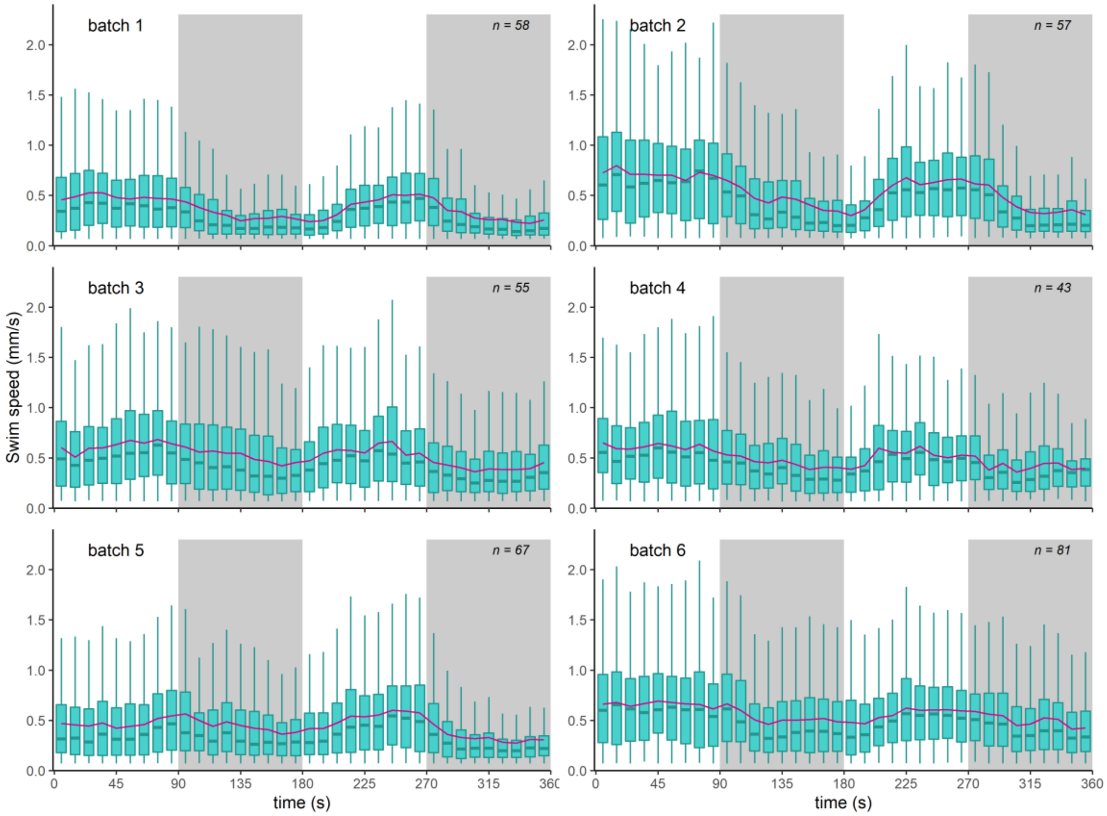

Fig. S5. Swimming speed data (mm s^{-1}) for six batches of larvae in the vertical free-swimming cuvette experiment. Data are represented as boxplots of the interquartile range with median line and whiskers extending to the most extreme values within 1.5× the interquartile range. Purple line represents mean swimming speed. White areas represent time periods with the light stimulus being on, whereas shaded boxes show the dark time periods.

Table S4. Wilcoxon rank sum statistical tests (W) comparing swimming speed (mm s^{-1}) in the middle 30 sec of both dark and the second light periods for the vertical cuvette free-swimming experiments. Columns 2 and 3 are median swimming speed values for each light condition. The bottom row shows the test result when data from all batches were pooled together.

batch	dark_med	light_on_med	W-value	p-value	signif.
1	0.175	0.375	1396172	2.59 e-119	***
2	0.252	0.535	982012	9.15 e-71	***
3	0.313	0.531	1527658	1.43 e-36	***
4	0.325	0.537	754550	2.89 e-31	***
5	0.248	0.442	1753602	2.68 e-46	***
6	0.355	0.537	2545960	2.67 e-38	***
**pooled**	**0.266**	**0.482**	**52652240.5**	**1.47 e**-296	**pooled**

Journal of Experimental Biology • Supplementary information

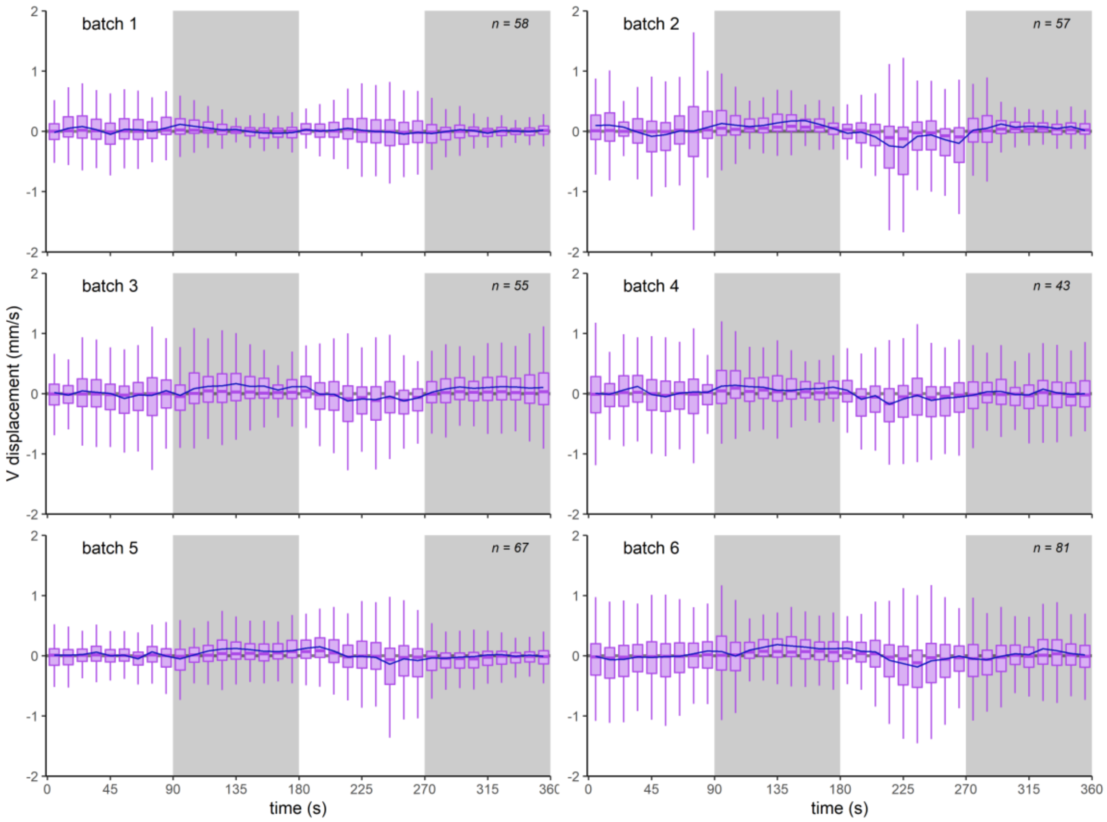

Fig. S6. Vertical displacement data (mm s^{-1}) for six batches of larvae in the vertical free-swimming cuvette experiment. Data are represented as boxplots of the interquartile range with median line and whiskers extending to the most extreme values within 1.5× the interquartile range. Dark blue line represents mean swimming speed. White areas represent time periods with the light stimulus being on, whereas shaded boxes show the dark time periods.

Table S5. Wilcoxon rank sum statistical tests (W) comparing vertical displacement (mm s^{-1}) in middle 30 sec of both dark and the second light periods for the vertical cuvette free-swimming experiments. Positive median values in columns 2 and 3 represent movement toward the light source on one side and negative values represent movement away from it. The bottom row shows the test result when data from all batches were pooled together.

batch	dark_med	light_on_med	W-value	p-value	signif.
1	0.011	0.011	2325504	0.170	-
2	0.060	-0.091	2118636	1.48 e-76	***
3	0.027	-0.085	2563379	1.91 e-51	***
4	0.019	-0.067	1221317	9.83 e-20	***
5	0.007	-0.010	2524931	6.66 e-05	***
6	0.042	-0.063	4121012	5.28 e-62	***
**pooled**	**0.025**	**-0.040**	**87668662**	**3.29 e**-151	***

Journal of Experimental Biology • Supplementary information

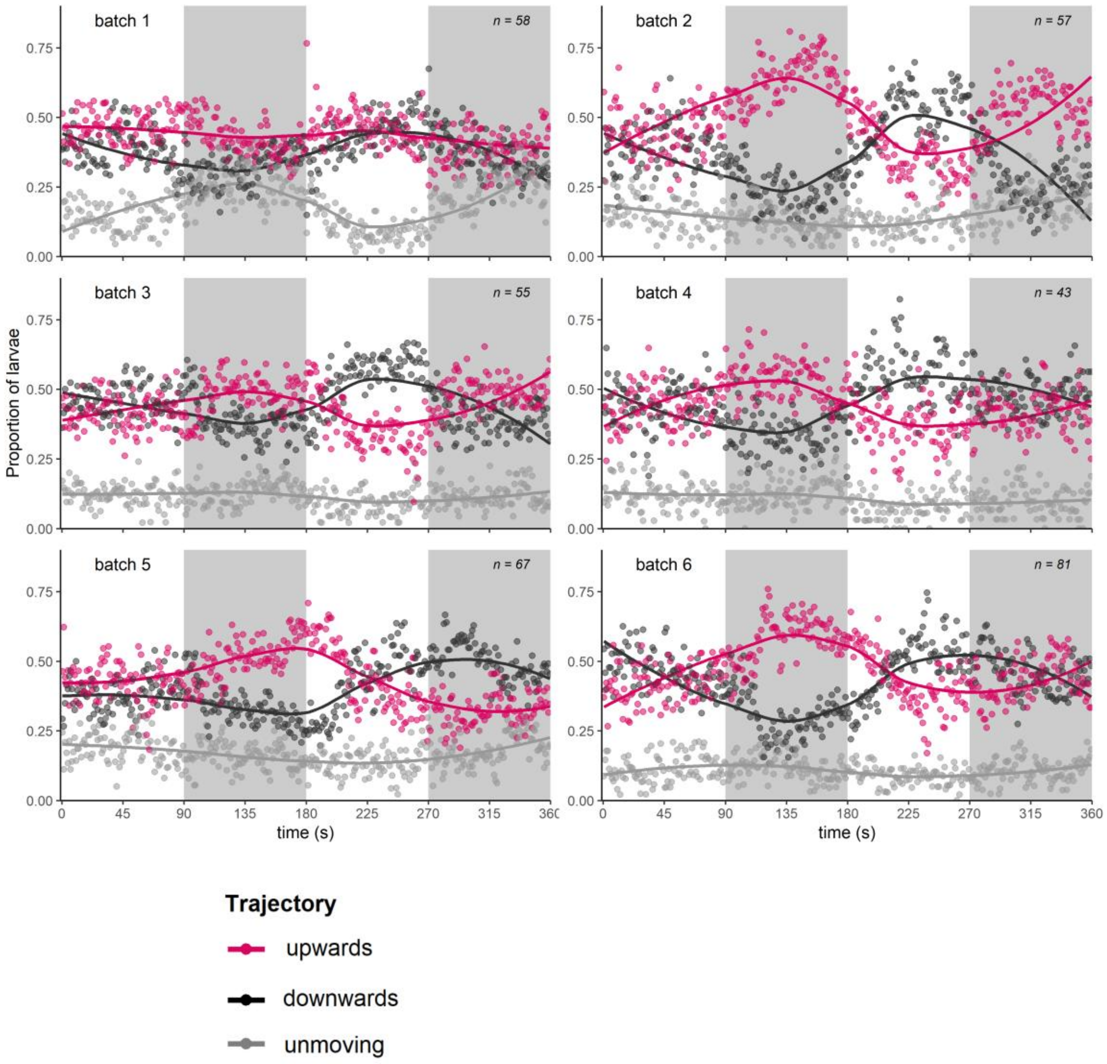

Fig. S7. Swimming trajectory data for six batches of larvae in the vertical free-swimming cuvette experiment. Data are represented as the proportion of total larvae at each time point that travelled upwards (red), downwards (black) or neither (grey) at each timepoint. Local regression curves fitted over points using LOESS-smoothing. White areas represent time periods with the light stimulus being on, whereas shaded boxes show the dark time periods.

Journal of Experimental Biology • Supplementary information

Video data supporting this manuscript are available online at: (Brodrick et al., 2025).

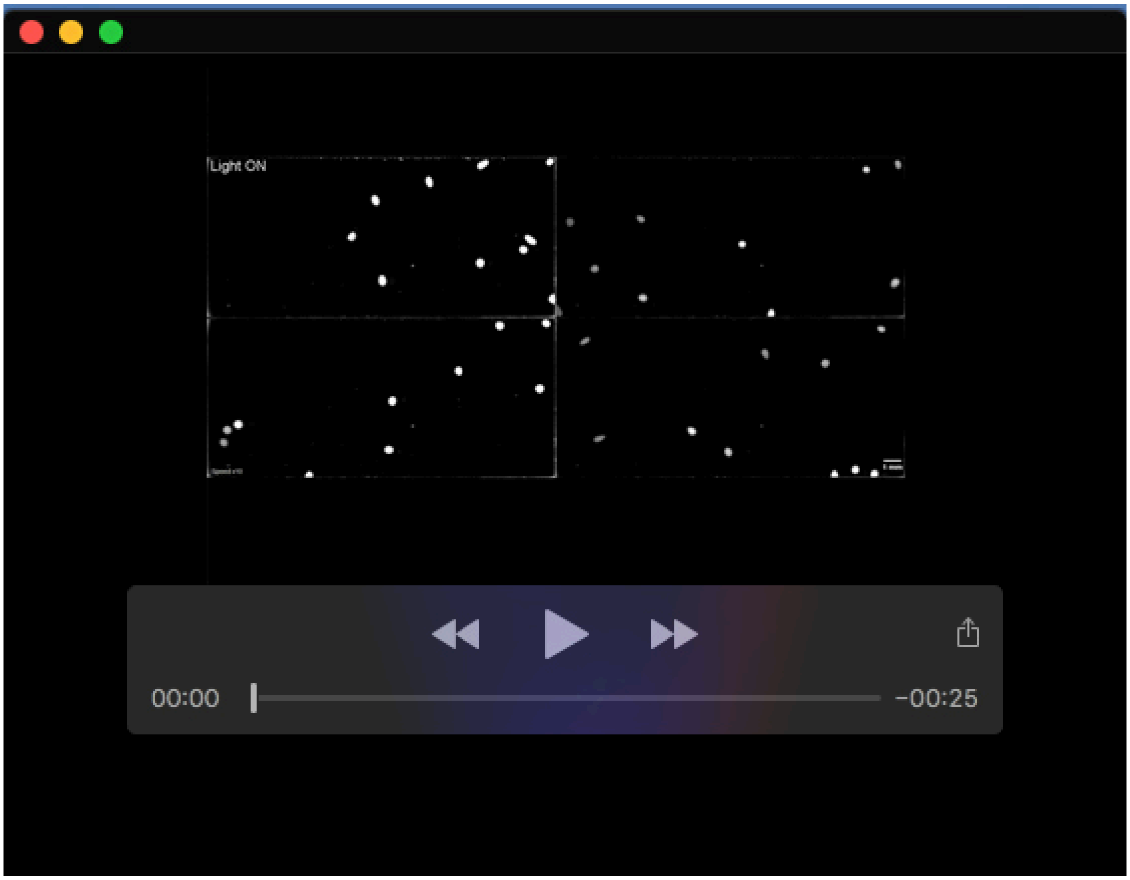

Movie 1. Horizontal tracking of coral larva trajectories in shallow cuvette. The video is split into four panels to show each of the 90 s light-on and dark phases. Playback is sped up by 10x. Tracks are coloured by larva ID and persist for each light phase showing the total trajectory. Light-on periods are characterized by faster swimming, covering greater distances in the cuvette. During dark periods, the larvae often tended to spin on the spot.

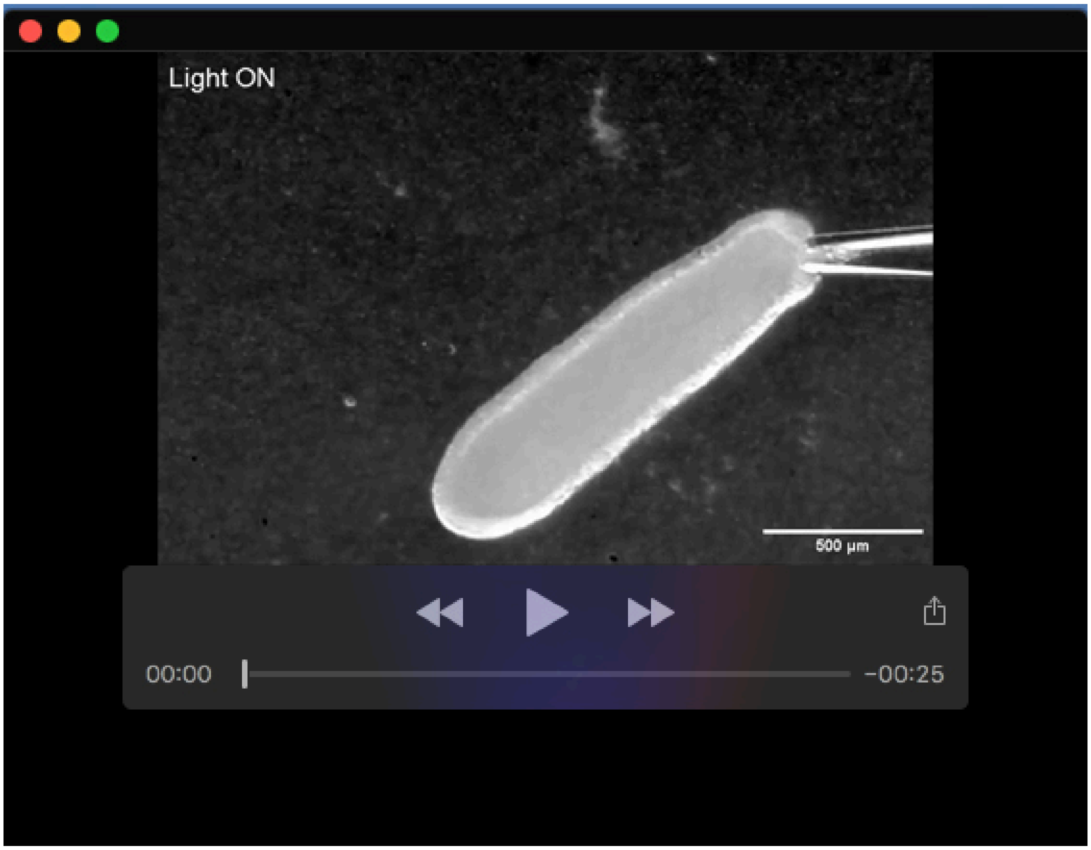

Movie 2. Tethered larva performing its light-off response to sudden intensity dimming. The larvais held in seawater containing nanoparticle beads to allow measurement of flow speeds via PIV. The first 5 s of the video show the larva elongated with beating ectodermal cilia under bright white light. At 5 s, the bright light is dimmed leaving the larva in dim red illumination only, which prompts the response of contracting the body into a round ball, and later, all flow stops around the larva as a result of ciliary arrest. Playback is in real time.

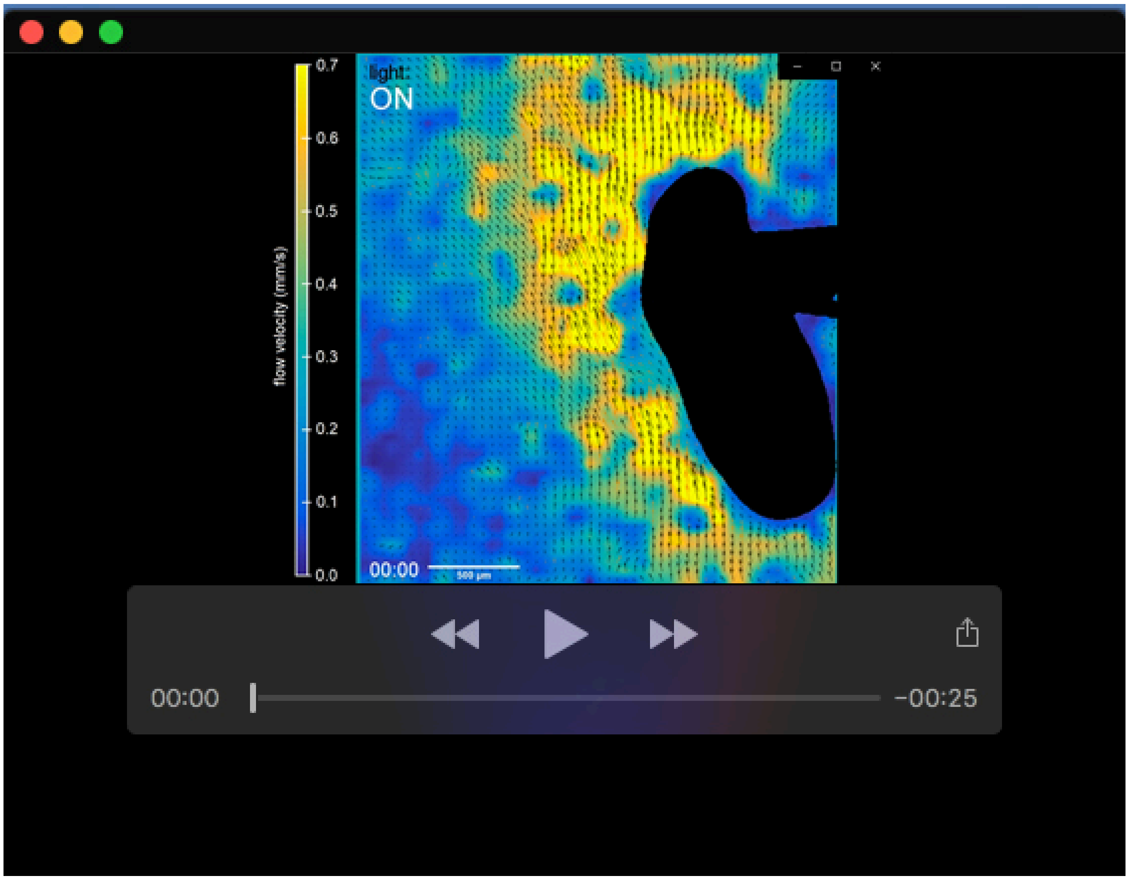

Movie 3. Particle image velocimetry (PIV) analysis. Example of a tethered larva (shaded black) with flow velocity in the surrounding seawater represented by arrows and colour (scale on left). The white light stimulus is on when the video starts but is dimmed 5 s later. All flow stops in the water surrounding the larva around 15 s after light dimming, suggesting ciliary arrest.

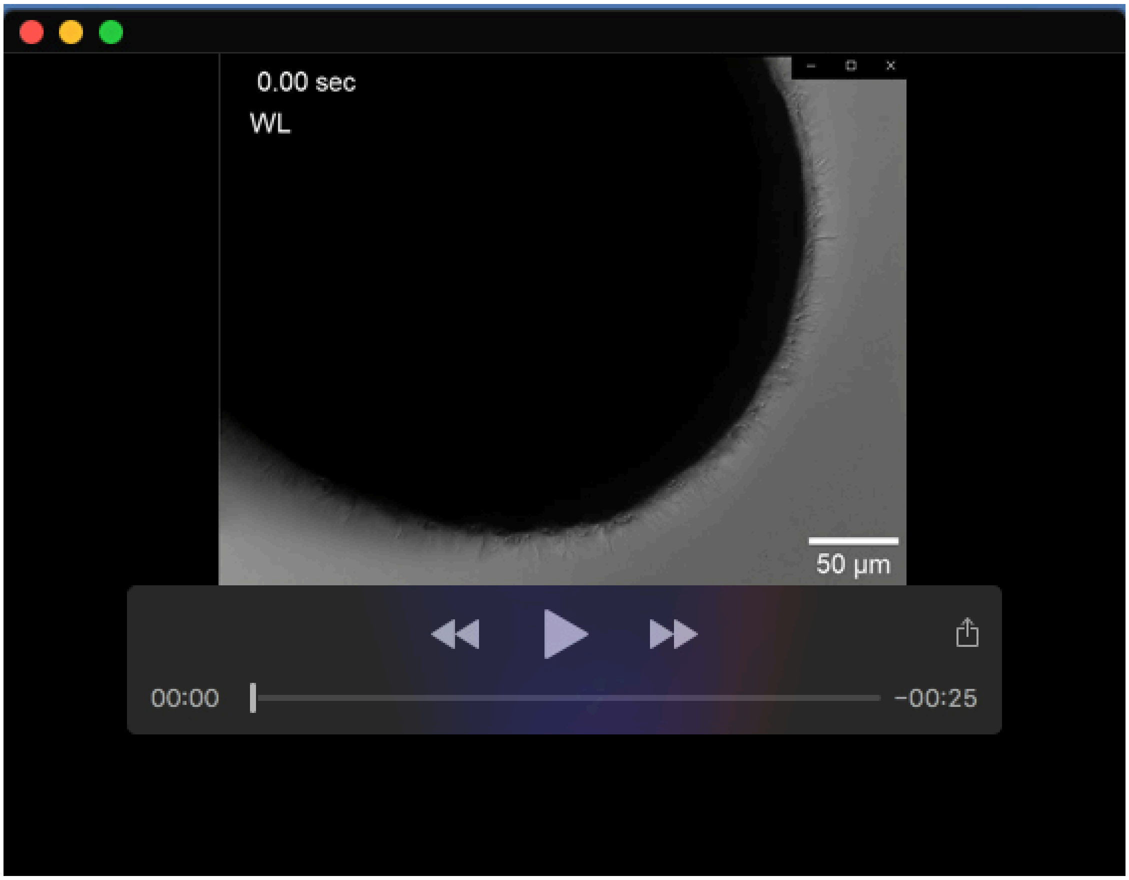

Movie 4. Ectodermal ciliary arrest of larva #1 in response to white light dimming. The video begins with white light (WL) on, then at 33.4 s, the white light is dimmed, and imaging continues using dim red illumination only (RL). An arrest of beating can be seen among the cilia in focus 4 s later (at 38 s). Video playback is in real time, but it has been compressed (from 500 fps) to 25 fps.

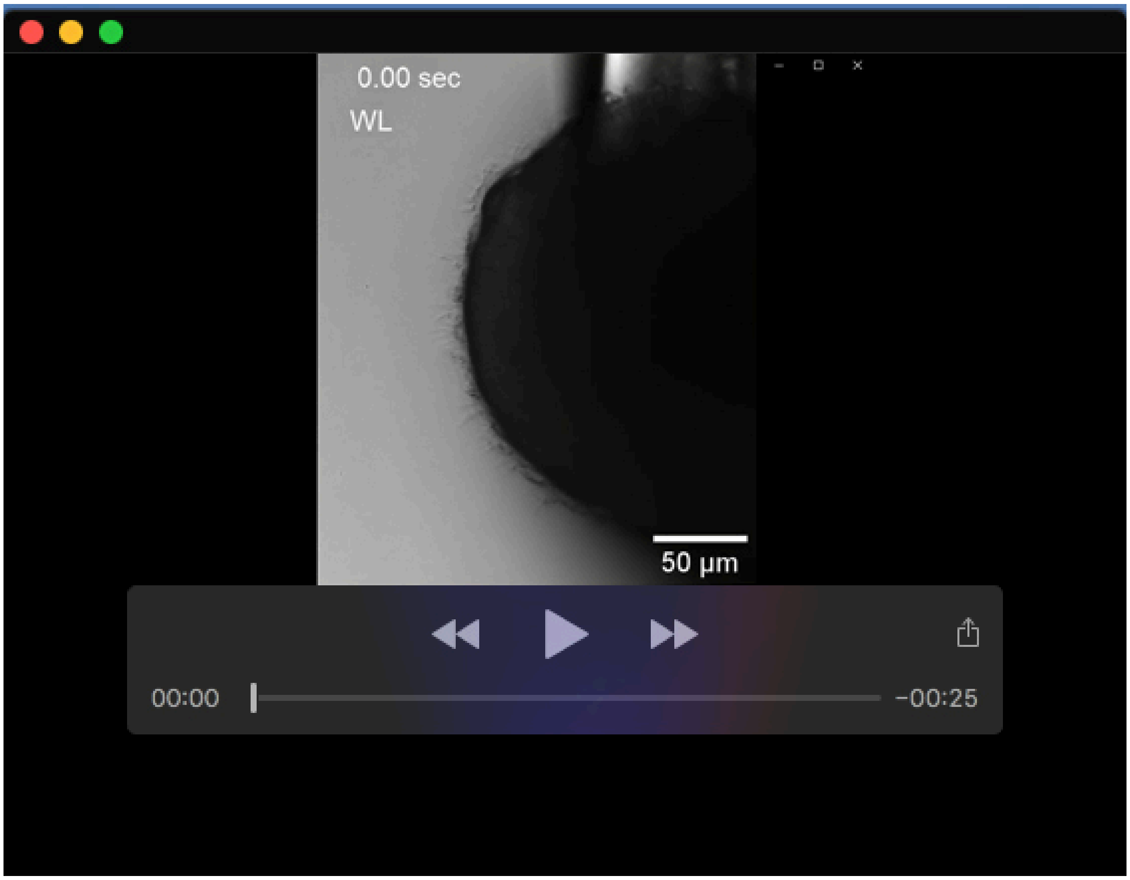

Movie 5. Ectodermal ciliary arrest of larva #2 in response to white light dimming. The video begins with white light (WL) on, then at 33 s, the white light is dimmed, and imaging continues using dim red illumination only (RL). An arrest of beating can be seen spreading down through the cilia in focus, starting at 77 s (43 s after the light dimming). Video playback is in real time, but it has been compressed (from 500 fps) to 25 fps.

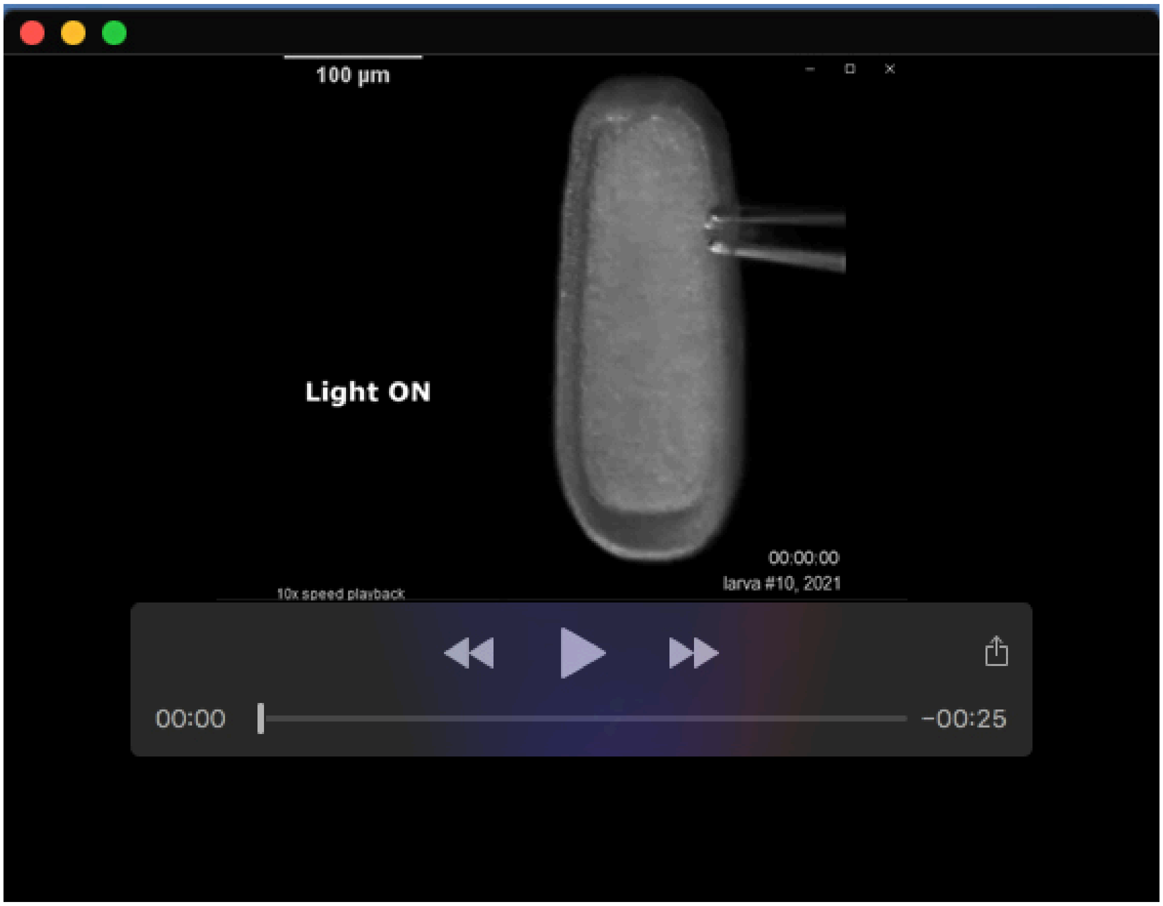

Movie 6. Coral larvae contract the body in response to light dimming. Two examples of coral larvae are shown from cohorts from consecutive spawning years**.** The larvae are tethered on a suction micropipette as the light stimulus is turned on and off, twice. Video playback is 10x. Note the muscle contraction events in dark periods, gradually rounding the body shape. An elongated shape is resumed in bright light periods via muscle relaxation.

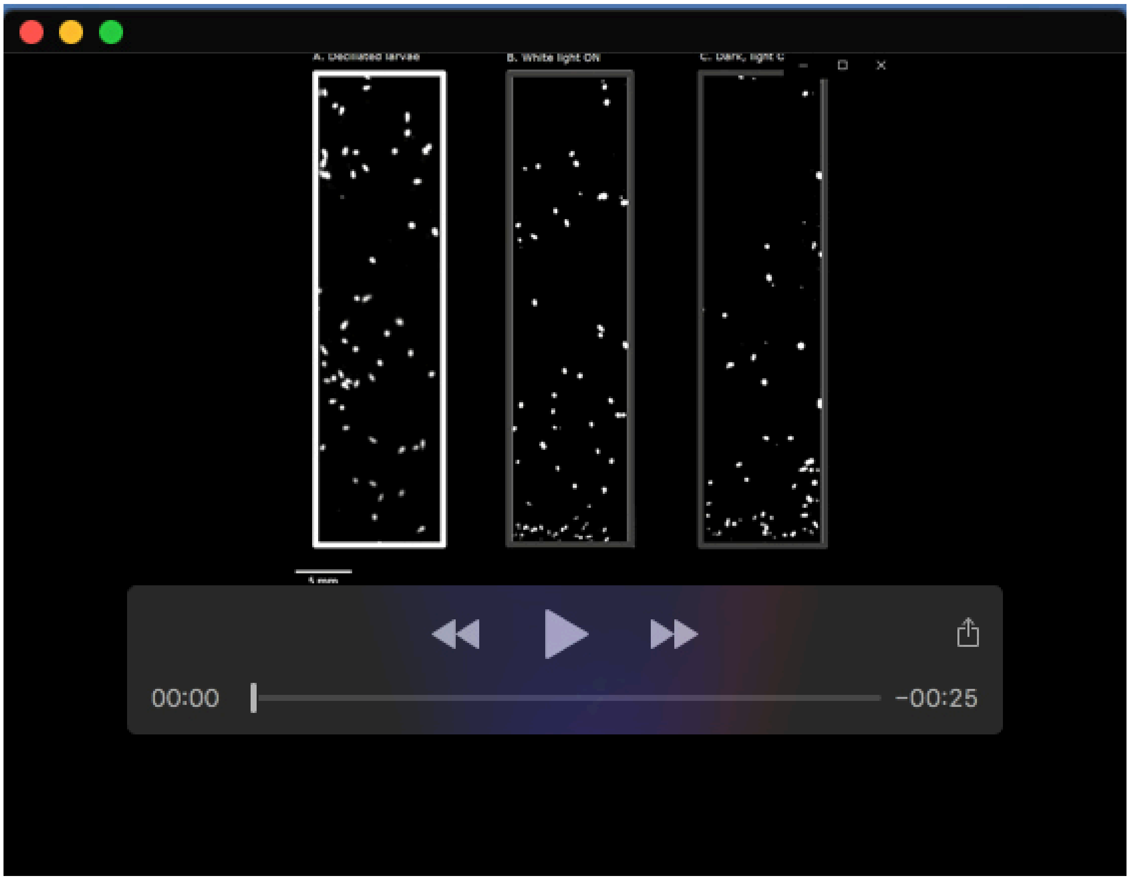

Movie 7. Vertical tracking of larval trajectories in tall cuvette. Fading trajectory tails span 30 frames and are coloured according to mean track speed (blue represents slow travel, while red is fast). Video playback is sped up by 10x. **A)** A batch of deciliated and immobile larvae float upwards over 50 s after mixing to disperse them in the column. **B)** Larvae swimming actively with white light on. Magenta box in the second repeat of the video highlights the larvae probing the base of the cuvette. **C)** Same batch of larvae as in B, in darkness after the sudden dimming of white light. Yellow box in second video repeat highlights that most larvae near the bottom surface showed alight-off response and floated very slightly upward. Whereas larvae higher in the column tended to continue active swimming as before.
